# The Importance of the Proper Definition of Adulthood: What is and What is Not Included in a Scientific Publication

**DOI:** 10.21470/1678-9741-2016-0049

**Published:** 2017

**Authors:** Luiz Fernando Canêo, Rodolfo Neirotti

**Affiliations:** Instituto do Coração do Hospital das Clínicas da Faculdade de Medicina da Universidade de São Paulo (InCor-HCFMUSP), São Paulo, SP, Brazil; Clinical Professor of Surgery and Pediatrics, Emeritus Michigan State, Brookline, MA, USA and honorary member of Sociedade Brasileira de Cirurgia Cardiovascular (Brazilian Society of Cardiovascular Surgery), São Paulo, SP, Brazil

**Dear Editor**,

We have read with great interest the study by Khan et al.^[1]^: “Surgery for Tetralogy of Fallot in Adults: Early Outcomes”, published in the Brazilian Journal of Cardiovascular Surgery, volume 31, issue 4, pages 300-3.

While the authors should be congratulated for their efforts to develop a pediatric cardiac surgery program in a difficult context, we have some comments, concerns, and questions around this article.

According to the references of this article, there are a few publications reporting the primary surgical treatment of Tetralogy of Fallot (TOF) during adulthood. In fact, only two publications about TOF in adults are enumerated in the references and some important reports are missing, including one from Brazil^[2]^.

This manuscript describes a single center experience in primary repair of adults with TOF in Pakistan during a two-year time-frame. They reported an impressive number of patients, but surprisingly they included patients between 12 and 43 years in this study.

The point at which a person progresses from childhood into adulthood may vary according to different cultures and the legal definition usually fluctuates between 16 and 21 years. Including younger patients increases the quantity, but using precise information is required in order to accurately evaluate surgical results and individualize risk factors.

As Lord Kelvin - an Irish mathematical physicist and engineer 1824-1907 - aptly put it, “What is not defined cannot be measured. What is not measured cannot be improved. What is not improved always deteriorates.” Then, it is imperative to define the beginning of adulthood to avoid confounding and to facilitate the application of methods for meaningful comparison of hospital mortality and morbidity for patients undergoing surgery for congenital heart disease.

According to the World Health Organization (WHO), an adult is a person older than 19 years of age unless national law delimits an earlier age, and an adolescent someone aged 10 to 19 years^[3]^. Additional literature retrieval about this issue, including those listed by the authors, shows that TOF in adults is commonly defined as subjects older than 18 years of age. Therefore, efforts should be made to embrace international parameters in our clinical practice.

In sum, it is not only a semantic issue, because there are potential errors in analyzing these data that include subjects younger than 18 years old named as “adults”. For instance, transannular patches are required more often in children than in adults, due to the more favorable anatomy encountered in adulthood^[2]^. In table 1, Khan et al.^[1]^ also showed a significant influence of age on the frequency in both right ventricular outflow tract and transannular patches.

Postoperative pulmonary valve insufficiency is a common sequela of transannular patches. Since pulmonary insufficiency is poorly tolerated in adults, some centers with experience in dealing with congenital heart disease in this age group are advocating placing a bioprosthetic valve in those patients requiring a transannular patch^[4]^. Khan et al.^[1]^ reported 25% of moderate/severe postoperative pulmonary regurgitation in their study. In this context, how did they evaluate these patients? How did they follow them? What did they do with them?

Even though repairing TOF in adult patients is not a common procedure nowadays due to the movement of cardiac surgery to the very young, it is very important for all of us to understand the outcomes related to their treatment. How many of these patients were truly adults? Knowing the number of patients below 18 years of age would answer this important question.

Age definition is a central issue, thus it may not be appropriated to use adults both in the title and as a keyword for this publication. A word of caution for all of us, a proper review of the manuscript could have avoided observations like this.

**Luiz Fernando Canêo**, MD, PhD - Instituto do
Coração do Hospital das Clínicas da Faculdade de Medicina da
Universidade de São Paulo (InCor-HCFMUSP), São Paulo, SP,
Brazil.**Rodolfo Neirotti**, MD, PhD, MPA, FEACTS - Clinical Professor of Surgery
and Pediatrics, Emeritus Michigan State, Brookline, MA, USA and honorary member of
Sociedade Brasileira de Cirurgia Cardiovascular (Brazilian Society of Cardiovascular
Surgery), São Paulo, SP, Brazil.
